# TELE-SAVVY@HOME: PRODUCING AND TESTING A FULLY ASYNCHRONOUS ONLINE TELE-SAVVY PROGRAM

**DOI:** 10.1093/geroni/igad104.0463

**Published:** 2023-12-21

**Authors:** Fayron Epps, Carolyn Clevenger, Melinda Higgins, Kenneth Hepburn

**Affiliations:** Emory University, Atlanta, Georgia, United States; Nell Hodgson Woodruff School of Nursing, Emory University, Atlanta, Georgia, United States; Emory University, Atlanta, Georgia, United States; Emory University, Atlanta, Georgia, United States

## Abstract

Group-based psychoeducational programs like the Savvy Caregiver program and its synchronous/asynchronous online adaptation, Tele-Savvy, have demonstrated that the acquisition of skills, knowledge, and caregiving mastery can ameliorate caregiving stress and enhance the quality of life for persons living with dementia. Many factors, including the constant presence of care recipients, preclude caregivers attending such programs, including synchronous online programs, and limit programs’ accessibility and availability. This presentation describes the development of a fully asynchronous online Savvy program (Tele-Savvy@Home; TS@H) and the feasibility, acceptability, and preliminary efficacy of the online program. TS@H employed online continuing education methods to present ~16 hours of content in 46 short interactive segments of 8-15 minutes each. TS@H pilot program participants had no interaction with other participants and didn’t engage with a live instructor. Pilot results provided preliminary confirmation of the pilot study’s primary and secondary hypotheses regarding positive benefits to caregivers and care recipients. Results from the 29 participants who completed the pilot program and provided follow-up data indicated the program produced moderate to large effects by end of study on enhancing caregiving mastery (d=0.645) and decreasing reported care recipient behavior (d=-0.834) and reduced caregiver reactivity to that behavior (d=-0.732). The program also produced small to moderate benefits (d = -0.25 to -0.49) in the areas of caregiver depression, anxiety, and burden. Caregivers reported the program helped them be less resentful of their caregiver role and to accept the changes in relationship dynamics with their person. These results will inform the next iteration of Tele-Savvy@Home.

